# HSP90 recognizes the N-terminus of huntingtin involved in regulation of huntingtin aggregation by USP19

**DOI:** 10.1038/s41598-017-13711-7

**Published:** 2017-11-01

**Authors:** Wen-Tian He, Wei Xue, Yong-Guang Gao, Jun-Ye Hong, Hong-Wei Yue, Lei-Lei Jiang, Hong-Yu Hu

**Affiliations:** State Key Laboratory of Molecular Biology, CAS Center for Excellence in Molecular Cell Science, Shanghai Institute of Biochemistry and Cell Biology, Chinese Academy of Sciences, University of Chinese Academy of Sciences, 320 Yueyang Road, Shanghai, 200031 P. R. China

## Abstract

Huntington’s disease (HD) is caused by aberrant expansion of polyglutamine (polyQ) in the N-terminus of huntingtin (Htt). Our previous study has demonstrated that HSP90 is involved in the triage decision of Htt, but how HSP90 recognizes and regulates Htt remains elusive. We investigated the interaction between HSP90 and the N-terminal fragments of Htt (Htt-N), such as the N-terminal 90-residue fragment (Htt-N90). Our results showed that HSP90 binds to the N-terminal extreme of Htt-N in a sequence just ahead of the polyQ tract. Structural integration of the middle and C-terminal domains of HSP90 is essential for interacting with Htt-N90, and the dimerization mediated by the C-terminal domain facilitates this interaction. Moreover, ubiquitin-specific protease 19 (USP19), a deubiquitinating enzyme interacting with HSP90, up-regulates the protein level of Htt-N90 and consequently promotes its aggregation, whereas disruption of the interaction between Htt-N90 and HSP90 attenuates the effect of USP19 on Htt-N90. Thus, HSP90 interacts with Htt-N90 on the N-terminal amphipathic α-helix, and then recruits USP19 to modulate the protein level and aggregation of Htt-N90. This study provides mechanistic insights into the recognition between HSP90 and the N-terminus of Htt, and the triage decision for the Htt protein by the HSP90 chaperone system.

## Introduction

**Figure 1:**
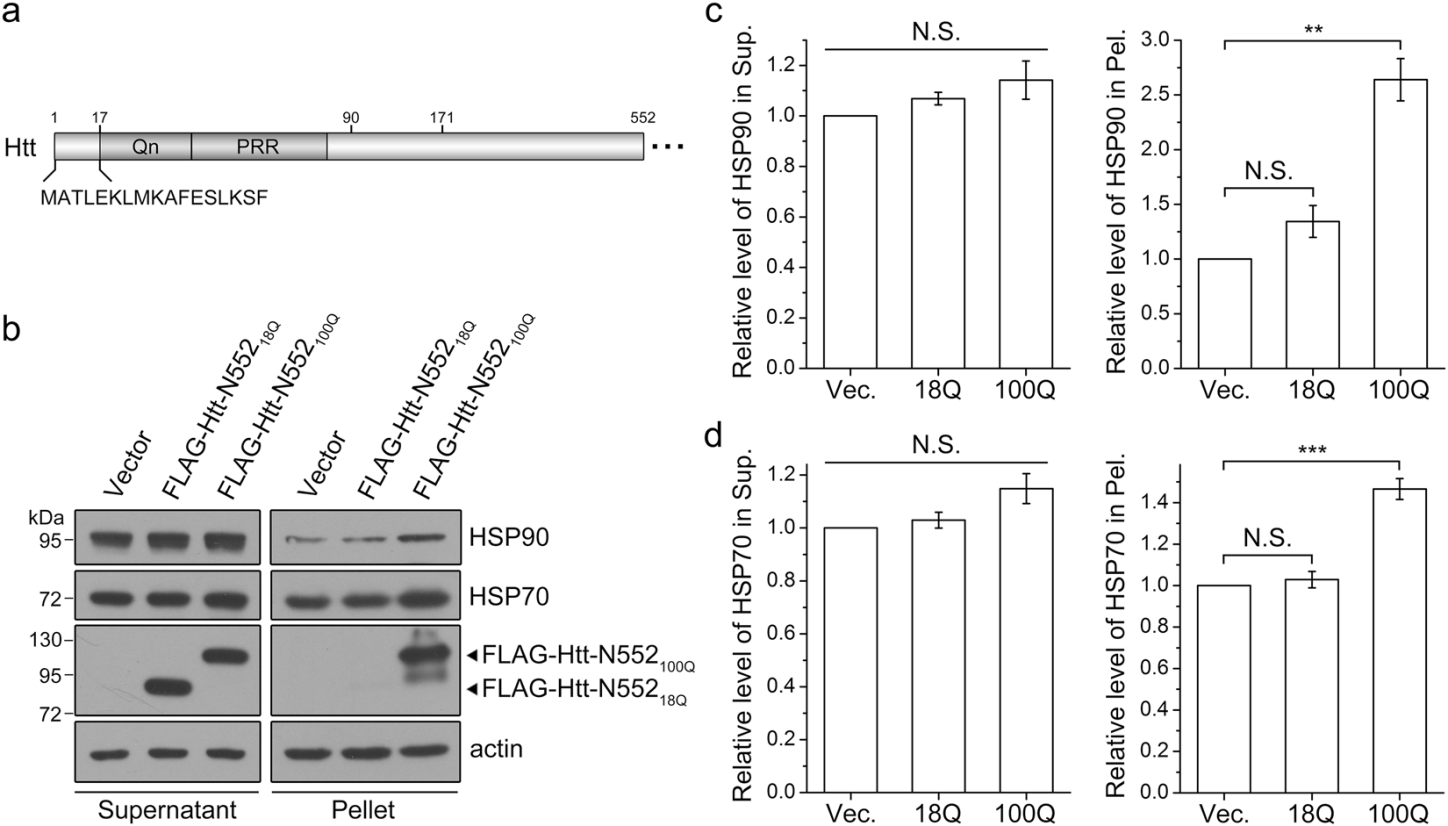
Supernatant/pellet fractionation showing that polyQ-expanded Htt sequesters chaperones into insoluble aggregates. (**a**) Domain architecture of the N-terminal fragment of Htt. The N-terminal 17-residue region is shown in detail. Qn, polyQ tract; PRR, proline-rich region. The normal 23 glutamines are included for numbering. (**b**) Sequestration of endogenous HSP90 and HSP70 by Htt-N552_100Q_. FLAG-tagged Htt-N552_18Q_ or Htt-N552_100Q_ was transfected into HEK 293 T cells, and the cell lysates were subjected to supernatant/pellet fractionation and Western blotting with anti-FLAG, anti-HSP90 and anti-HSP70 antibodies. (**c**,**d**) Quantification of the amounts of endogenous HSP90 (**c**) or HSP70 (**d**) in supernatant and pellet fractions. Data were from (**b**) and presented as Mean ± SEM (n = 3). **p < 0.01; ***p < 0.001; N.S., no significance. Vec., vector; Sup., supernatant; Pel., pellet.

Proteins with an aberrant expansion of polyglutamine (polyQ) are the main pathological causes of nine neurodegenerative diseases^1–4^. Among them, Huntington’s disease (HD) is caused by a CAG trinucleotide repeat expansion within the first exon of *huntingtin* gene^5^. The corresponding translated protein huntingtin (Htt) contains a polyQ tract in its N-terminus. In the normal population, the length of polyQ tract ranges from about 11 to 34, whereas the pathologic polyQ stretch expands to over 35 repeats^6^. PolyQ-expanded Htt is aggregation-prone and tends to form cytoplasmic and nuclear inclusion bodies, which are associated with cellular toxicity, neuron degeneration and pathogenesis^7^. A variety of N-terminal fragments of Htt (Htt-N) have been demonstrated in mouse model of HD^8^. Most of these fragments are derived from proteolysis of full-length Htt protein^9–12^. The smallest N-terminal fragment that has been found to be pathologic is the N-terminal 90-residue fragment (Htt-N90), which is generated by aberrant splicing of *Htt* exon 1^13^. Htt-N90 consists of an N-terminal 17-residue sequence, a polyQ tract, and a proline-rich region (PRR) in the C-terminus (see Fig. 1a). It has been reported that polyQ-expanded Htt-N90 is sufficient to cause a progressive neurological phenotype in mice^14^. Moreover, many studies have revealed that the N-terminal fragments containing expanded polyQ are prone to aggregation and contribute to Htt-induced cytotoxicity^15–17^. Meanwhile, the shorter N-terminal fragments containing the polyQ tract may be more toxic than the longer ones^18,19^. Due to their crucial role in HD pathogenesis, polyQ-expanded N-terminal fragments of Htt have been widely used in the related studies.

To maintain proteostasis and prevent diseases, the organisms have established a subtle protein quality control (PQC) system, in which molecular chaperones are the hub of this system. Heat shock protein 90 (HSP90) is a highly conserved molecular chaperone that is present from bacteria to mammals^20^. HSP90 is a dimeric protein and each monomer has three domains, N-terminal domain (HSP90-N), middle domain (HSP90-M) and C-terminal domain (HSP90-C). HSP90-N is involved in nucleotide binding and hydrolysis^21^, HSP90-M mainly mediates client protein interactions^22^, and HSP90-C is responsible for dimerization^23^. As the hub of proteostasis, HSP90 plays an important role in triage decision of diverse client proteins that are key players in many biological processes^20^. However, the underlying mechanisms for the interaction and recognition of HSP90 with its client proteins are still elusive. Accumulating evidence suggests that HSP90 prefers to recognize intrinsically unstable proteins and bind the hydrophobic region of client proteins^24–26^. Moreover, the binding site for the clients on HSP90 is obscure. Besides the middle domain, the N- and C-terminal domains have also been reported for interacting with its clients^27–29^. So, it seems that the interacting domains on HSP90 vary with different clients.

PolyQ-containing proteins have propensities to misfolding and readily expose their hydrophobic residues, thus they are potential candidates for HSP90 clients. It has been reported that Htt associates with HSP90 and inhibition of HSP90 leads to the degradation of Htt^30^. However, the molecular basis for the interaction between HSP90 and Htt is enigmatic. Also, how HSP90 participates in the stabilization and degradation of Htt needs to be clearly elucidated. Our previous study has demonstrated that cytoplasmic ubiquitin-specific protease 19 (USP19), through interacting with HSP90, up-regulates the protein levels of the N-terminal 552-residue fragments of Htt (Htt-N552) with normal and expanded polyQ, and consequently increases the aggregates formed by polyQ-expanded Htt-N552^31^. Here, we investigated the interactions between HSP90 and the N-terminal fragments of Htt by biochemical and biophysical approaches. We found that HSP90 is able to bind the N-terminal amphipathic α-helix of Htt and this interaction contributes to modulating the protein level and aggregation of Htt by USP19. This study provides some clues to the mechanism underlying HSP90-Htt recognition, and corroborates the previous finding that USP19 regulates the client protein Htt through cooperation with the HSP90 chaperone system.

## Results

### PolyQ-expanded Htt sequesters HSP90 into insoluble aggregates

Previously, various cellular essential proteins have been found in the aggregates formed by polyQ-expanded proteins such as Htt^32–34^. Here, we applied the N-terminal 552-residue fragment of Htt (Htt-N552) as a polyQ protein to investigate whether polyQ-expanded Htt sequesters endogenous HSP90 chaperone into aggregates. We transfected FLAG-tagged Htt-N552_18Q_ and Htt-N552_100Q_ respectively into HEK 293 T cells and performed supernatant/pellet fractionation experiment (Fig. 1). As the previous report that the polyQ-expanded Htt fragments readily form insoluble aggregates^33^, Htt-N552_100Q_ exhibited a considerable amount of aggregates in pellet fraction, whereas Htt-N552_18Q_ mostly remained in supernatant. Upon over-expression of Htt-N522, the supernatant fraction showed a slight increase of endogenous HSP90 as compared with that of the vector control, but there was no significant difference in statistical analysis. In contrast, HSP90 in the pellet fraction increased significantly when over-expression of Htt-N552_100Q_ but not Htt-N552_18Q_ (Fig. 1b,c). A similar result was also obtained from detecting distribution of another chaperone HSP70, showing that Htt-N552_100Q_ sequestered more endogenous HSP70 into aggregates than Htt-N552_18Q_ (Fig. 1b,d). These results demonstrate that polyQ-expanded Htt can sequester the HSP90 and HSP70 chaperones into insoluble aggregates^35^, implying that the N-terminal fragments of Htt can be recognized by HSP90 as well as HSP70.

### HSP90 interacts with Htt on the N-terminus

**Figure 2:**
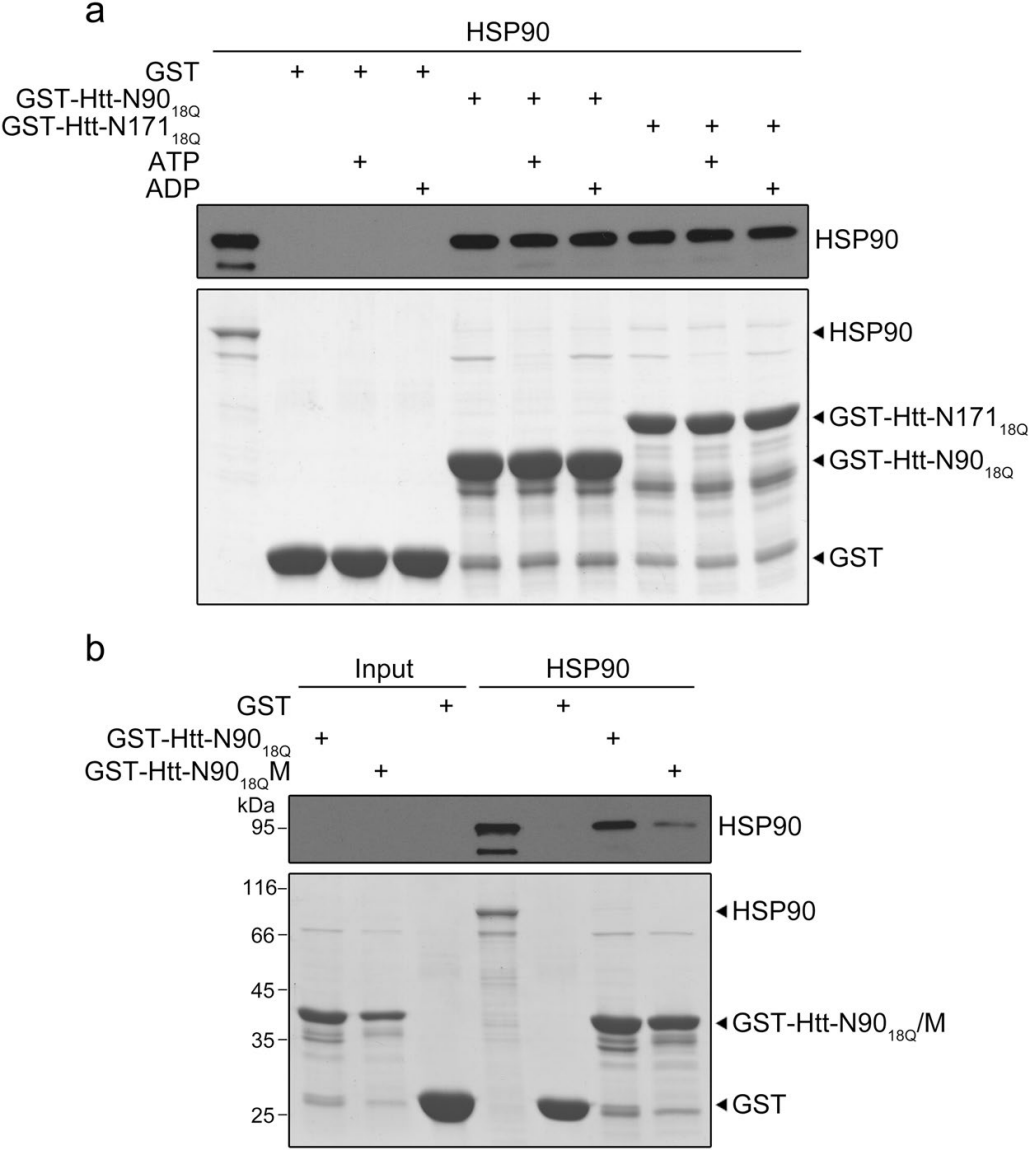
The N-terminal fragments of Htt interact with HSP90. (**a**) GST pull-down experiment for the interactions of Htt-N90_18Q_ or Htt-N171_18Q_ with HSP90 in the presence of ATP or ADP. Bottom panel, SDS-PAGE with Coomassie blue staining; top panel, Western blotting to detect HSP90 with an anti-HSP90 antibody (5% input). (**b**) Examination of the interaction of Htt-N90_18Q_ or its F11A/L14A mutant (Htt-N90_18Q_M) with HSP90. HSP90 was detected by Western blotting with the anti-HSP90 antibody (top panel). M, mutant.

To better understand the mechanism underlying the sequestration of HSP90 by Htt-N552_100Q_, we used GST pull-down experiment to investigate the interactions between the N-terminal fragments of Htt and HSP90. We constructed two GST-fused N-terminal fragments, Htt-N90_18Q_ and Htt-N171_18Q_. Probably due to the weak or transient interaction, we did not clearly detect pronounced HSP90 pulled down by the Htt-N fragments with Coomassie blue staining, so we resorted to Western blotting with an anti-HSP90 antibody to detect the pull-down products (Fig. 2). The result showed that both Htt-N90_18Q_ and Htt-N171_18Q_ could interact with HSP90 (Fig. 2a), whereas mutation of the key residues significantly attenuated the interaction (Fig. 2b) (see below). As known, nucleotide binding or ATP hydrolysis may induce conformational change of HSP90, and this process is essential for maturation of the client proteins^36^. So we wondered whether the nucleotide binding state influences the interactions of the N-terminal fragments with HSP90. The pull-down data showed that Htt-N90_18Q_ and Htt-N171_18Q_ could interact with HSP90 no matter ATP/ADP was present or not (Fig. 2a). Thus, HSP90 interacts with and recognizes Htt on the N-terminal region, being independent of nucleotide binding and ATP hydrolysis.

### The N-terminal extreme of Htt is essential for interacting with HSP90

**Figure 3:**
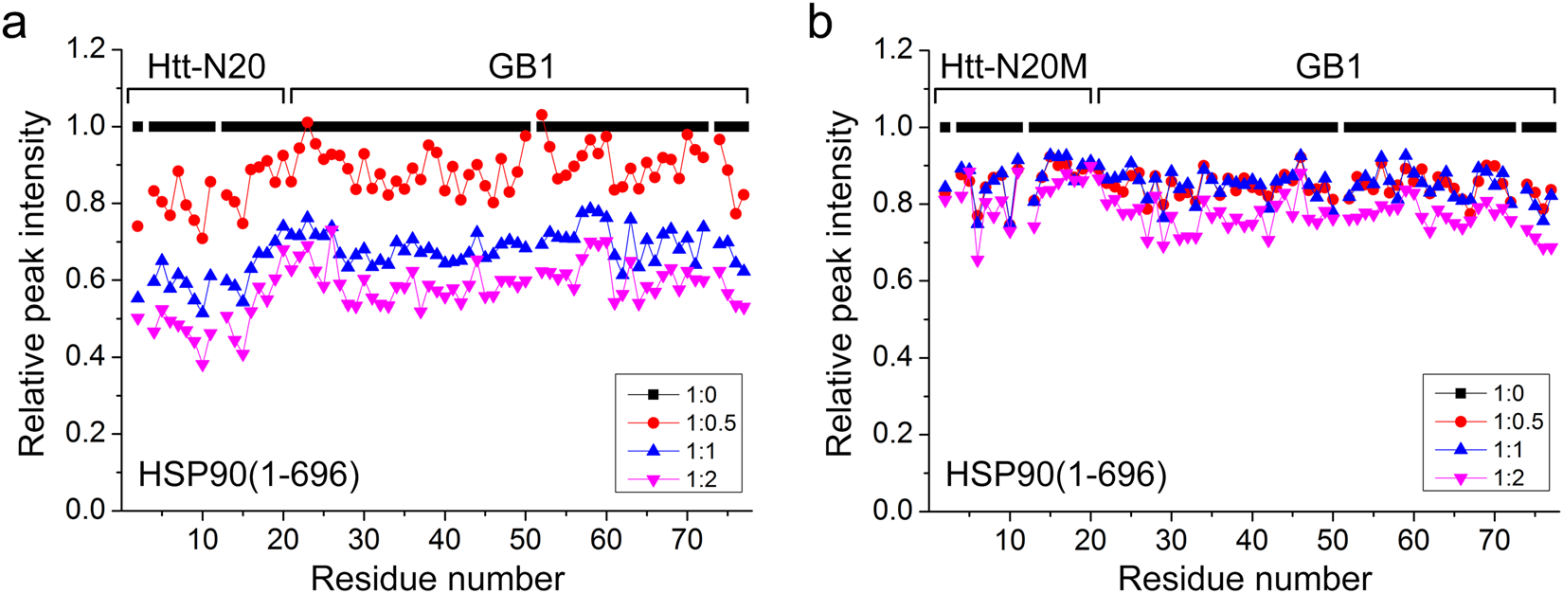
NMR titration showing the specific interaction between Htt-N20 and HSP90. (**a**) Plot of the relative peak intensities of amides against the residue number of C-terminal GB1-fused Htt-N20 upon titration with HSP90(1–696). The peak intensities were normalized as 1 for all peaks of free Htt-N20-GB1 except those of unassigned residues. (**b**) As in (**a**), the F11A/L14A mutant (Htt-N20M-GB1) upon titration with HSP90(1–696).

It was previously reported that HSC70 bound to the N-terminal 17-residue region of Htt^37^. To gain the information of HSP90 recognizing its clients, we further investigated their interactions by NMR techniques. We selected an N-terminal 20-residue fragment and fused GB1 at the C terminus (Htt-N20-GB1)^38^, and performed NMR titration on the interaction of ^15^N-labeled Htt-N20-GB1 with HSP90(1–696). The HSQC spectra of ^15^N-labeled Htt-N20-GB1 were obtained in the absence or presence of HSP90(1–696) (Supplementary Fig. S1). With titration of HSP90(1–696), the resonance peaks of Htt-N20-GB1 became weak gradually. Based on the titration data, we generated a profile of the relative peak intensities of amides against the residue number of Htt-N20-GB1 upon titration with HSP90(1–696) with molar ratios of 1: 0.5, 1: 1, and 1: 2 (Fig. 3a). The peak intensities of the residues in Htt-N20 significantly decreased with addition of HSP90(1–696). It is noteworthy that the peak intensities of GB1, regarded as a well negative control, also decreased but with less extent as compared with those of Htt-N20. This is because, as in the case of the integrated fusion protein, the molecular tumbling of the GB1 moiety was also perturbed by binding with HSP90(1–696). We also performed titration with HSP70 as a positive control to examine its interaction with Htt-N20 under the same conditions. Similarly, HSP70 titration caused a remarkable decrease of the peak intensities in the Htt-N20 moiety as compared with those in the GB1 region (Supplementary Fig. S2). These data further demonstrate that Htt-N20 is able to interact with HSP90 as well as HSP70.

To reconfirm the observation, we performed a transferred NOE (TrNOE) experiment^39,40^ on the chemically synthetic peptide Htt-N20 in the presence of HSP90(1–696). The NOESY spectrum of the Htt-N20 peptide exhibited a number of transferred NOE peaks with addition of one-twentieth aliquot of HSP90(1–696) (Supplementary Fig. S3), suggesting that Htt-N20 does interact with HSP90 albeit with a quite weak affinity.

### The N-terminal extreme of Htt forms an amphipathic α-helix for HSP90 binding

**Figure 4:**
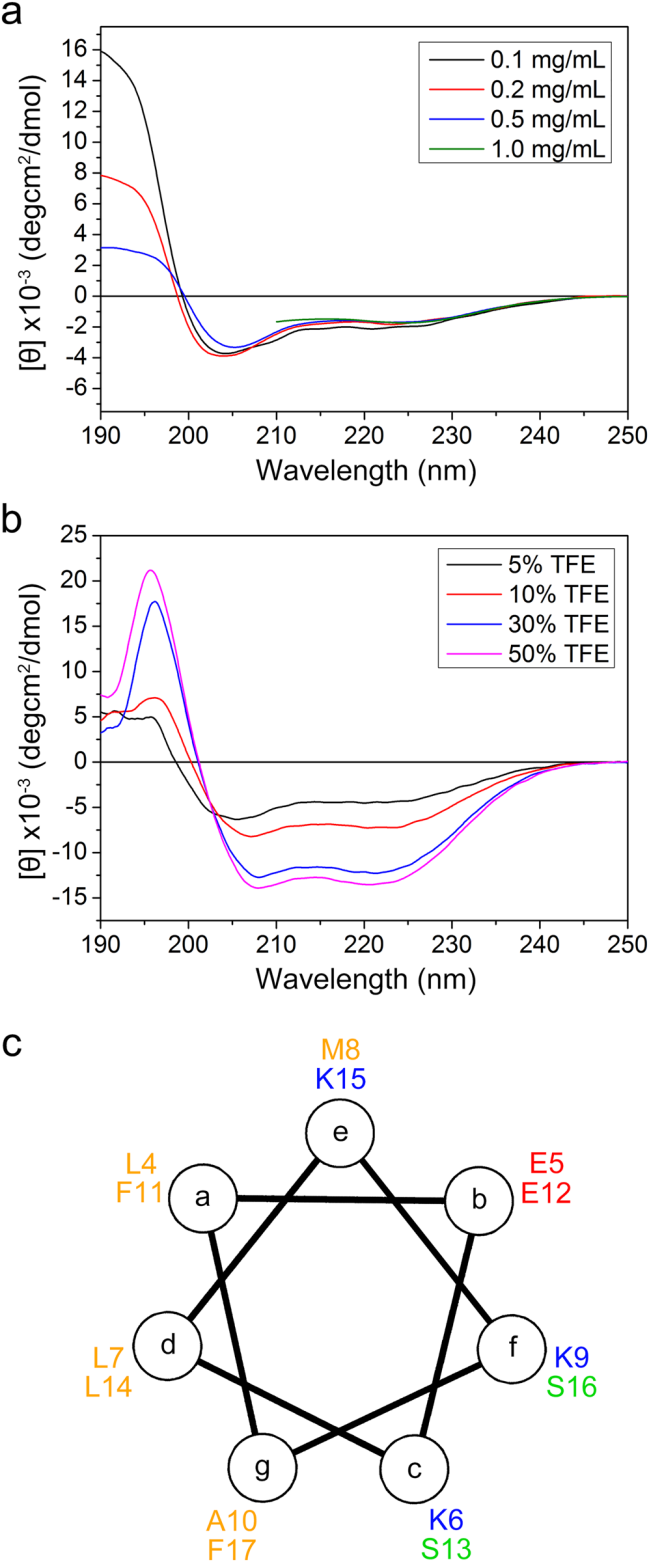
CD spectra exhibiting formation of an amphipathic α-helix in the N-terminal extreme. (**a**) Far-UV CD spectra of Htt-N20 with various concentrations. A cuvette with 1-mm path-length was used for recording at different concentrations, but for a concentration of 1.0 mg/mL, the CD signals at wavelengths below 210 nm were not recorded due to noise. (**b**) CD spectra of Htt-N20 in different concentration of TFE (v/v). The concentration of Htt-N20 peptide was 0.2 mg/mL. deg, degree. (**c**) The helical wheel plot for residues 4–17 of Htt-N20. The hydrophobic residues are shown in yellow, serines in green, glutamates in red, and lysines in blue.

We attempted to get insights into the detailed molecular mechanism by which Htt-N20 interacts with HSP90. Previous studies have demonstrated that the N-terminal 17-residue region forms an amphipathic α-helical dimer^37,41^. We characterized the secondary structure of the Htt-N20 peptide by circular dichroism (CD) spectroscopy (Fig. 4a, Supplementary Fig. S4). The CD spectra exhibited almost similar at different concentrations with a relatively strong negative peak at around 204 nm and a shoulder at 222 nm, indicating that Htt-N20 formed a marginal α-helical structure, but not a helical coiled-coil dimer, where the spectrum was recorded in a very high peptide concentration^37^. Note that the positive peaks below 200 nm varied dramatically at different concentrations (Fig. 4a), possibly due to the increase of noise in this wavelength region at high peptide concentrations. We also investigated the α-helix formation induced by trifluoroethanol (TFE) as monitored by CD spectroscopy^42^. With the increase of TFE concentration, the α-helix contents of Htt-N20 obviously increased, shown as appearance of the double negative peaks at 222 nm and 208 nm in the spectra (Fig. 4b). This result suggests that the Htt-N20 peptide has a high propensity to form α-helix. The helical wheel plot showed that the N-terminal extreme (residues 4–17) of Htt formed an amphipathic α-helix with the hydrophobic residues resided on one side (Fig. 4c).

**Figure 5:**
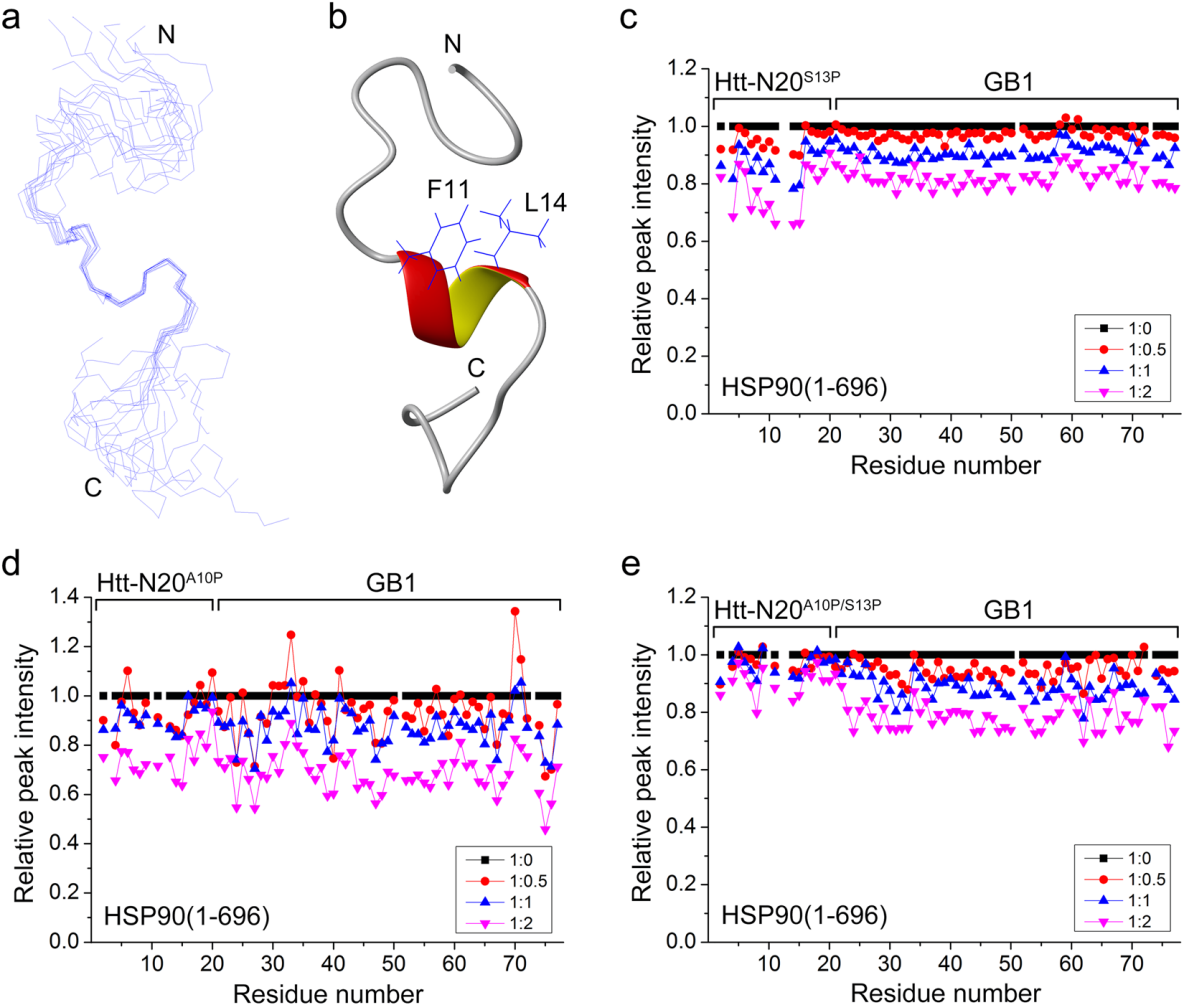
Solution structure of Htt-N20 and NMR titration for the helical mutants. (**a**) Superposition of the backbone traces of the 10 lowest-energy structures. (**b**) Ribbon diagram of a representative structure of the Htt-N20 peptide. The structure was solved in a C-terminal GB1-fused peptide (Htt-N20-GB1). (**c**) Plot of the relative peak intensities of amides against the residue number of Htt-N20^S13P^-GB1 upon titration with HSP90(1–696). The average of the intensities for residues 4–17 was ~0.86 at a 1:1 ratio titration. (**d**) As in (**c**), Htt-N20^A10P^-GB1. (**e**) As in (**c**), Htt-N20^A10P/S13P^-GB1.

We then solved the solution structure of Htt-N20 in a C-terminal GB1-fused peptide (Htt-N20-GB1) by NMR techniques^43^. The experimental restraints and structural statistics of Htt-N20 are summarized in Supplementary Table S1. While most of the residues in Htt-N20 are highly flexible, a short sequence of Phe11 - Leu14 forms a one-turn amphipathic α-helix (Fig. 5a,b). In the structural view, the two hydrophobic residues (Phe11, Leu14) aforementioned do reside on one side of the helix. To ask whether this short helix is required for HSP90 binding, we introduced a Pro residue into the helical region (A10P, S13P), which was supposed to disrupt the helical structure. The average of the relative peak intensities of the Htt-N20 moiety for the S13P mutant was ~0.86 at a 1:1 ratio titration (Fig. 5c), whereas that for the wild type was ~0.59 (Fig. 3a). Similarly, the A10P mutation reduced its binding affinity with HSP90 significantly (Fig. 5d), especially the double mutation (A10P/S13P) almost abolished the interaction (Fig. 5e). Thus, the NMR titration on the mutants suggests that this amphipathic α-helix is important for Htt-N20 binding to HSP90.

Considering that HSP90 may bind to the hydrophobic surface of its client protein, we speculated that HSP90 is able to bind the hydrophobic side of the amphipathic α-helix of Htt-N20. There are four hydrophobic residues (Leu4, Leu7, Phe11 and Leu14) probably important for the interaction. Interestingly, the peak intensities of Phe11 and Leu14 decreased more robustly than those of other residues in Htt-N20 upon HSP90 titration (Fig. 3a). So, we introduced a double mutation F11A/L14A in the helical region and characterized its interaction with HSP90. Although not strictly quantitative, GST pull-down showed that wild-type Htt-N90_18Q_ could pull down a considerable amount of HSP90, but the F11A/L14A mutant (Htt-N90_18Q_M) could not (Fig. 2b). In NMR titration, the peak intensities of the F11A/L14A mutant (Htt-N20M) remained almost unchanged with addition of HSP90(1–696) (Fig. 3b), suggesting that the F11A/L14A mutation may disrupt the binding. Thus, the mutation analysis reconfirms the specific interaction between Htt-N20 and HSP90, and the hydrophobic residues like Phe11 and Leu14 contribute directly to their interaction. Taken together, these results demonstrate that the hydrophobic side of the amphipathic α-helix (Phe11 - Leu14) is essential for the interaction of the N-terminal Htt with HSP90.

### Both the middle and C-terminal domains of HSP90 are involved in the interaction with Htt-N20

**Figure 6:**
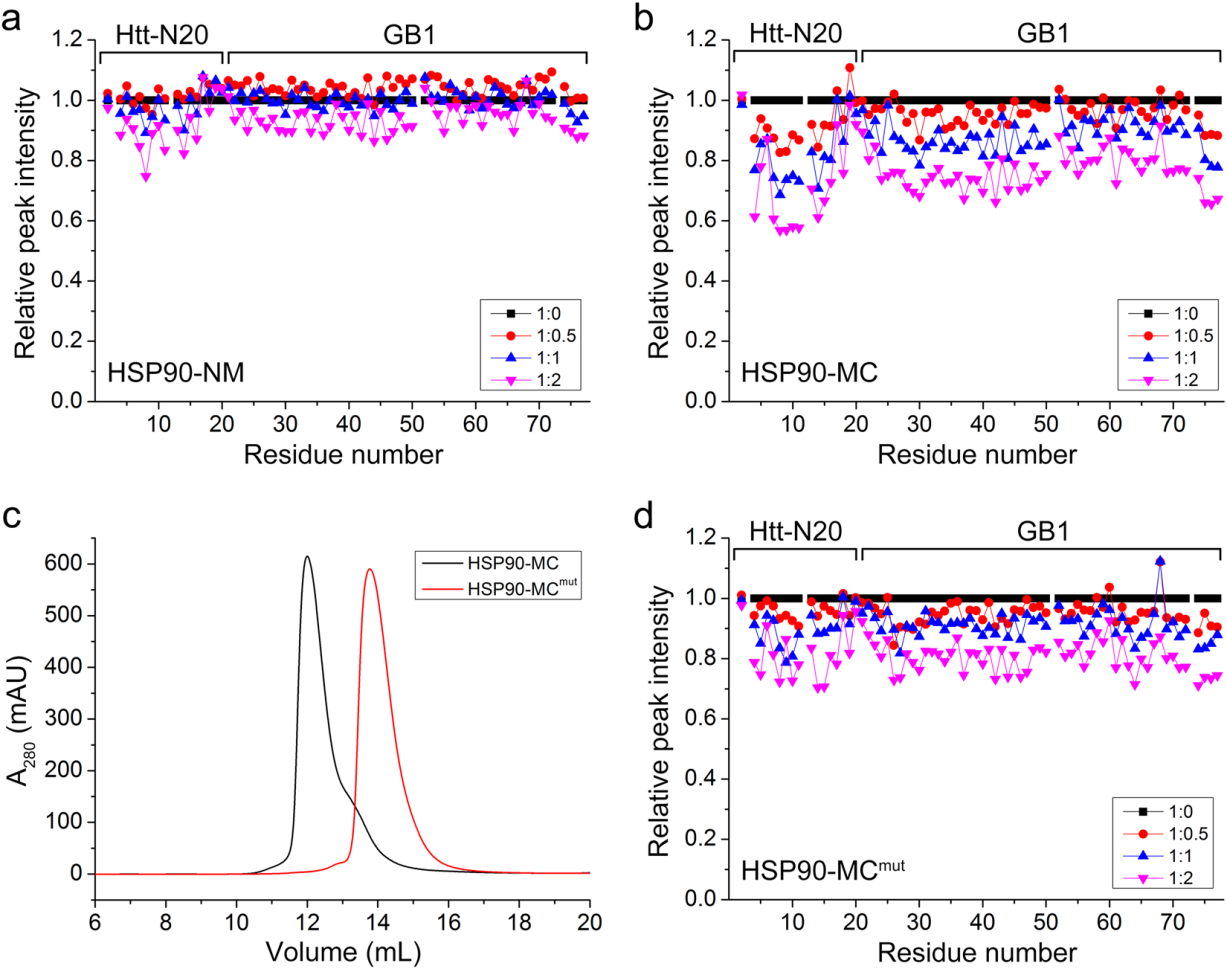
NMR titration suggesting that HSP90-MC is involved in the interaction with Htt-N20. **(a**,**b**) Plot of the relative peak intensities of amides against the residue number of Htt-N20-GB1 upon titration with HSP90-NM (residues 1–548) (**a**) or HSP90-MC (236–696) (**b**). (**c**) Elution profiles of HSP90-MC and its mutant (I688A/Y689A/I692A, HSP90-MC^mut^) by using size exclusion chromatography. Purified WT or mutant HSP90-MC protein (~100 μM) was loaded on a Superdex-200 Increase 10/300 GL column in a phosphate buffer (20 mM phosphate, 50 mM NaCl, pH 6.5). A_280_, Absorbance at 280 nm; mAU, milli absorbance unit. (**d**) As in (**a**), Htt-N20-GB1 upon titration with HSP90-MC^mut^.

To date, few studies have clarified the binding sites on HSP90 for client proteins. Different client proteins may interact with different domains of HSP90^44^. To characterize the binding domains of HSP90 for Htt-N20, we titrated ^15^N-labeled Htt-N20-GB1 with three separate domains of HSP90, respectively. These domains include HSP90-N (residues 1–235), HSP90-M (236–548) and HSP90-C (549–696). To our surprise, no obvious peak-intensity change was observed on Htt-N20-GB1 when it was titrated with each separate domain (Supplementary Fig. S5). Then we applied double-domain fragments of HSP90, HSP90-NM and HSP90-MC, in NMR titration. The result showed that HSP90-MC titration caused an intensity decrease of Htt-N20, whereas HSP90-NM did not (Fig. 6a,b). Notably, the most pronounced decrease of the peak intensities were from residues Leu4, Leu7 - Phe11 and Leu14. This also corroborates our previous observation that the hydrophobic side formed by Leu4, Leu7, Phe11 and Leu14 is involved in the interaction of Htt-N20 with HSP90. Because only HSP90-MC can interact with Htt-N20 in NMR titration, we supposed that combination of the middle and C-terminal domains or dimerization of the C-terminal domain may be essential for HSP90 interacting with Htt-N20. To address this issue, we prepared a three-point mutation (I688A/Y689A/I692A, HSP90-MC^mut^) on the H5 helix of the C-terminal domain that was previously demonstrated to contribute to the dimerization of HSP90^45^. We firstly analyzed effect of the mutation on the dimerization of HSP90-MC by size exclusion chromatography (SEC). The main peak of HSP90-MC was eluted at a volume of ~12 mL, whereas that of HSP90-MC^mut^ showed a higher elution volume of ~13.8 mL (Fig. 6c). Based on calibration in SEC profile, their apparent molecular weight was ~147 kDa for the wild type and ~73 kDa for the mutant, suggesting that HSP90-MC formed a dimer, whereas HSP90-MC^mut^ was a monomer in solution. Note that there is a shoulder in the HSP90-MC profile, indicating a small fraction of the monomers existed. Subsequently, we examined the interaction of HSP90-MC^mut^ with Htt-N20 by NMR titration. The result showed that the mutation attenuated the interaction with Htt-N20 considerably (Fig. 6d); despite the fact that the middle and C-terminal domains were still combined in a double-domain fragment. Based on the above observation, we propose that Htt-N20 interacts with HSP90 on the middle domain, and dimerization of the C-terminal domain is crucial for the middle domain integration and the interaction with Htt-N20.

### Modulation of Htt-N90 by USP19 is dependent on the interaction with HSP90

**Figure 7:**
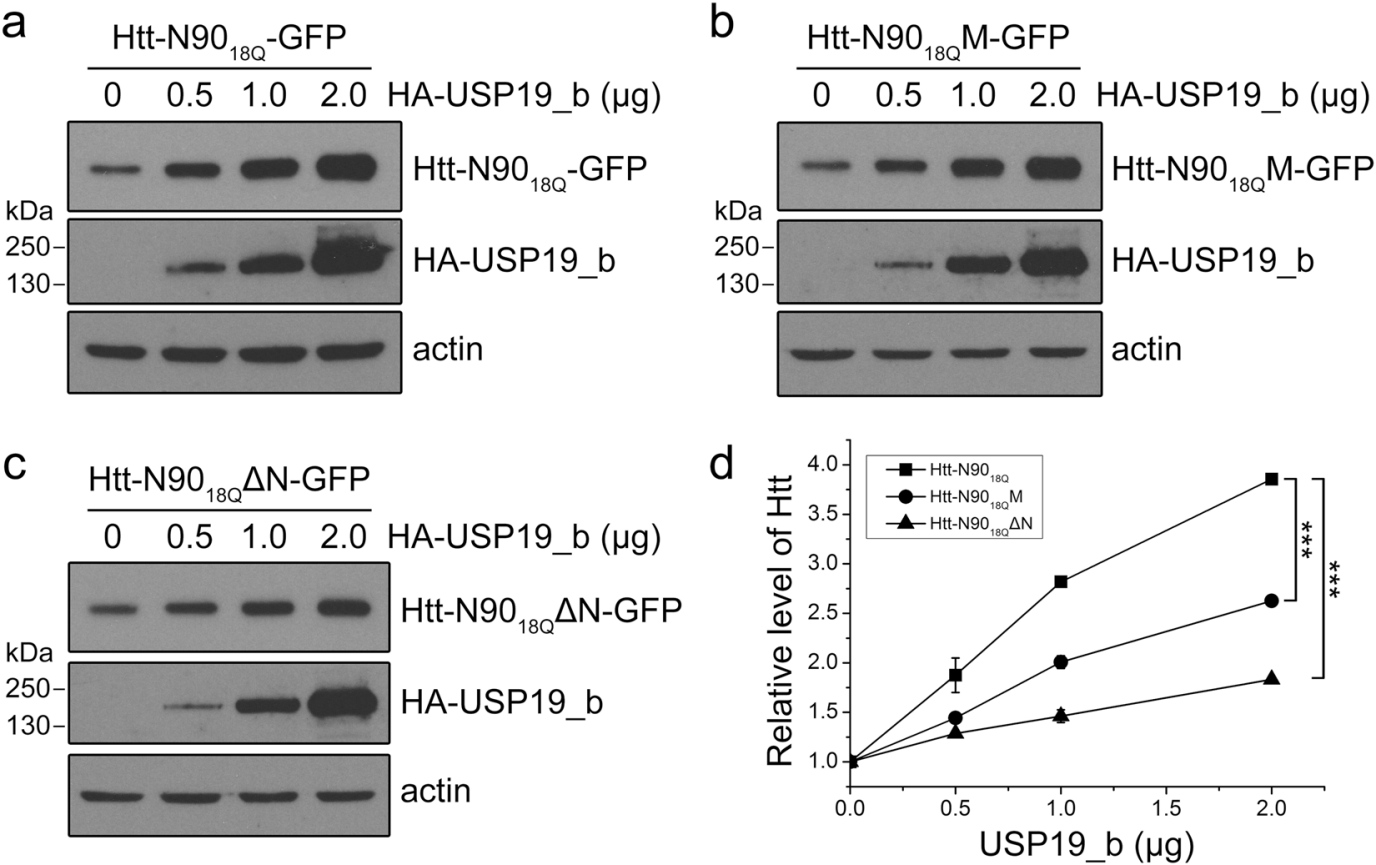
Interaction with HSP90 is essential for regulation of the protein level of Htt-N90_18Q_ by USP19_b. (**a**) Effect of USP19_b on the protein level of Htt-N90_18Q_ in a dose-dependent manner. Htt-N90_18Q_-GFP was co-transfected with different dose of HA-USP19_b into HEK 293 T cells. The amounts of Htt-N90_18Q_ were detected by Western blotting with an anti-GFP antibody. (**b**) As in (**a**), effect of USP19_b on the protein level of the F11A/L14A mutant (Htt-N90_18Q_M-GFP). (**c**) As in (**a**), effect of USP19_b on the protein level of the N-terminally 14-residue deleted mutant (Htt-N90_18Q_ΔN-GFP). (**d**) Quantification of the amounts of Htt-N90_18Q_-GFP and its mutants affected by USP19_b. Data were from (**a**), (**b**) and (**c**), and presented as Mean ± SEM (n = 3). ***p < 0.001.

As a client protein of HSP90, Htt is regulated by the HSP90 chaperone system in cell. We have previously reported that the cytoplasmic isoform of a deubiquitinating enzyme USP19 (USP19_b) cooperates with HSP90 and regulates the protein level and aggregation of Htt^31^. However, whether the regulatory function of USP19_b relies on the interaction between HSP90 and Htt is not clear. To address this question, we investigated the effect of USP19_b on the protein level and aggregation of Htt-N90. Because of the low-expression level of FLAG-tagged Htt-N90_18Q_ in cells, we fused GFP to Htt-N90_18Q_ at the C-terminus. Consistent with our previous observation, USP19_b up-regulated the protein level of wild-type Htt-N90_18Q_ in a dose-dependent manner (Fig. 7a,d), whereas it had less effect on the F11A/L14A mutant (Htt-N90_18Q_M) (Fig. 7b,d), especially on the N-terminal 14-residue deleted mutant (Htt-N90_18Q_ΔN) (Fig. 7c,d).

**Figure 8:**
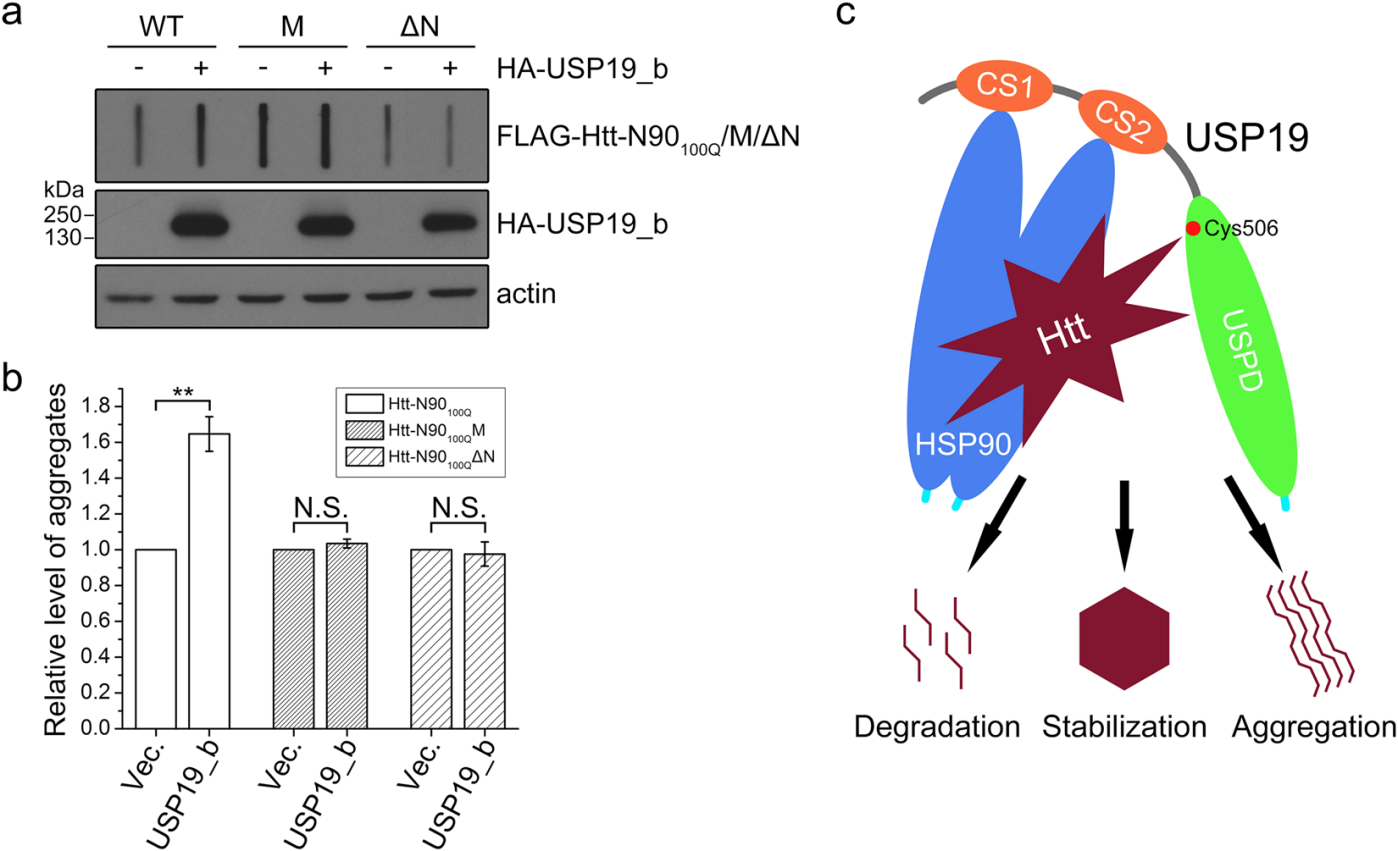
The promoting effect of USP19_b on the aggregation of Htt-N90_100Q_ is dependent on the HSP90-Htt-N interaction. (**a**) Effect of USP19_b on the aggregation of Htt-N90_100Q_ and its mutants. FLAG-tagged Htt-N90_100Q_ (WT), its F11A/L14A mutant (M) or N-terminally 14-residue deleted mutant (ΔN) was co-transfected with HA-USP19_b into HEK 293 T cells. The cell lysates were subjected to filter trap and Western blotting with an anti-FLAG antibody. (**b**) Quantification of the aggregates of Htt-N90_100Q_ and its mutants affected by USP19_b. Data were from (**a**) and presented as Mean ± SEM (n = 3). **p < 0.01; N.S., no significance. Vec., vector. (**c**) Schematic representation for recognition and triage decision of Htt modulated by USP19 through the HSP90 chaperone. HSP90 (blue) recognizes the client protein Htt (brown) and then recruits the deubiquitinating enzyme USP19 through the CS domains (orange) to regulate the ubiquitination state of Htt. These processes ultimately determine the triage of Htt for stabilization, aggregation or degradation.

USP19_b could promote the aggregation of polyQ-expanded Htt^31^. To verify the effect of HSP90-Htt-N interaction, we performed a filter trap experiment to detect the aggregates formed by polyQ-expanded Htt-N90. As expected, USP19_b could increase the aggregates of wild-type Htt-N90_100Q_ (Fig. 8a, 2^nd^ lane; Fig. 8b). However, when mutation of Phe11 and Leu14 (Htt-N90_100Q_M) or deletion of the N-terminal 14 residues (Htt-N90_100Q_ΔN), USP19_b lost its ability to increase the aggregates (Fig. 8a,b). These results suggest that the specific interaction between HSP90 and Htt-N90 is important for modulating the protein level and aggregation of Htt-N90 by USP19_b.

## Discussion

Htt is the causative protein of HD. A hallmark of HD pathogenesis is the accumulation of polyQ-expanded Htt aggregates, which result in cytotoxicity and neurodegeneration. To maintain proteostasis and defend against disease, HSP90 and other components of PQC system are recruited together to regulate the abnormal pathogenic proteins^46^. We investigated the interaction between HSP90 and its client protein Htt, and found that HSP90 interacts with the N-terminal extreme of Htt just ahead of the polyQ tract. We also demonstrated that the interaction between HSP90 and Htt-N90 is important for the regulation of Htt aggregation by a deubiquitinating enzyme USP19, supporting our model that USP19 modulates the client proteins through cooperating with HSP90^31^.

Previous studies have shown that the N-terminal segment of Htt may form an amphipathic α-helix^41,47,48^ and mediate the interaction of the N-terminus with chaperone HSC70^37^. Here, we demonstrate, for the first time, that HSP90 interacts with the N-terminus of Htt probably on the hydrophobic side of the amphipathic α-helix. Although the mutation in the N-terminal extreme of Htt-N90 disrupts its interaction with HSP90 in our GST pull-down experiment (Fig. 2b), we do not exclude the possibility that HSP90 may bind to other regions in the longer N-terminal fragments or intact Htt. Since HSP90 prefers hydrophobic interactions with its clients^49^, any hydrophobic region in Htt may be the potential binding site for HSP90. We think that, in the case of Htt-N90, its N-terminal extreme is an essential site for HSP90 binding. Considering that Htt-N90 is implicated in HD pathogenesis^14^, this specific interaction with HSP90 is important for quality control of the Htt protein by the HSP90 chaperone system. Moreover, a number of literatures have revealed that the N-terminal extreme facilitates the oligomerization and aggregation of Htt-N90 through its self-association^37,50–52^. However, the binding of HSC70 to the N-terminal extreme of Htt, competing against the intermolecular interactions, impedes formation of the oligomers and aggregates by Htt-N90^37^. Whether HSP90 has similar influence on the aggregation process of Htt-N90 needs further exploration.

We have demonstrated that the integrity of the middle and C-terminal domains of HSP90 is involved in the interaction with the N-terminal extreme of Htt. To our surprise, the three separate domains of HSP90 are not able to interact with Htt-N20. Dimerization by the C-terminal domain of HSP90 is probably required for the interaction. Moreover, because the separate C-terminal domain does not interact with Htt-N20 despite its ability to form a dimer, we suggest that the middle domain of HSP90 provides the major site for binding with the N-terminal extreme of Htt, while the C-terminal domain buttresses the dimeric form and steric orientation of the middle domain. Based on this point, we propose that the binding site for Htt-N20 probably resides on the internal moiety enclosed by the two subunits of the middle domain of HSP90 in the dimeric form. In the case of middle-domain monomer, its interaction with Htt-N20 may be too weak to be detected in NMR titration. Taken together, we raise a hypothesis that dimerization of the C-terminal domain of HSP90 allows two middle-domain subunits to form a closed conformation that creates a cleft between them. Htt-N20 binds to the interior cleft constructed by the two middle-domain subunits, and this structure stabilizes the HSP90-Htt-N complex and enhances their interactions. The studies on HSP90 binding to other client proteins, such as glucocorticoid receptor (GR) and staphylococcal nuclease (SN), also revealed the similar interaction pattern^29,53^. Nevertheless, more detailed structural information is needed to confirm the interaction pattern between HSP90 and Htt-N. Recently, many structure analysis methods including NMR, small-angle X-ray scattering (SAXS) and electron microscopy (EM), have been applied to study HSP90-client interactions^24,26–29,53–55^. For the sake of HSP90-Htt-N recognition, the structural information will deepen our understanding of the underlying mechanism by which HSP90 interacts with Htt and other pathogenic clients.

As a critical member of PQC system, HSP90 participates in the triage decision of numerous client proteins^20^. For polyQ-disease proteins, HSP90 decides their destiny for folding, aggregation or degradation. Once the aggregation-prone proteins fold incorrectly, they will form insoluble aggregates and accumulate in cell. During aggregation processes, their interacting partners such as HSP90 and other components of PQC system are sequestered into the aggregates and lose their normal functions, which consequently reduces the effective concentration of the active partners in soluble fraction^35^. The loss of available chaperones further exacerbates protein aggregation and causes collapse of proteostasis. A couple of studies have revealed that the sequestration of chaperones by aggregated polyQ-expanded proteins impairs their normal cellular function^56,57^. Therefore, chaperone dysfunction is also considered to be a vital cause of cytotoxicity and neurodegeneration^58^. We have demonstrated that polyQ-expanded Htt sequesters HSP90 and HSP70 into insoluble aggregates (Fig. 1). Whether the sequestration by Htt aggregates compromises functions of the chaperones is an interesting issue for future investigation.

To ameliorate the toxicity caused by Htt aggregates, two major degradation systems, ubiquitin-proteasome^59,60^ and autophagy-lysosome^61,62^, are devoted to clearance of the misfolded proteins. Meanwhile, chaperones are also contributable to degradation of the Htt protein. HSP90 and HSP70 promote misfolded Htt to undergo ubiquitination and proteasomal degradation through recruiting E3 ligases such as CHIP^63^ and parkin^64^. Our previous study has demonstrated that cytoplasmic USP19 deubiquitinates the Htt-N552 fragment and consequently increases the protein level and aggregation, and this effect is dependent on the interaction of USP19 with HSP90^31^. In this study, we have observed similar regulatory function of USP19 on the Htt-N90 fragment, and this effect is attenuated when the interaction between HSP90 and Htt-N90 has been disrupted. Based on these lines of evidence, we propose a model for triage decision of the Htt protein modulated by USP19 through HSP90 (Fig. 8c). HSP90 is responsible for recognition of the client protein Htt, then the deubiquitinating enzyme USP19 is recruited by HSP90 to regulate the ubiquitination state of Htt. HSP90 binds and recognizes normal Htt protein for assisting correct folding. A subset of Htt especially polyQ-expanded Htt that remains unfolded under chaperoning effect undergoes degradation pathway, which is well controlled by ubiquitin ligases and deubiquitinating enzymes associated with HSP90. In the case of misfolded Htt that has not been cleared out in time, the accumulated proteins form insoluble aggregates, sequester molecular chaperones and other interacting partners, and consequently result in cytotoxicity and neurodegeneration in HD pathogenesis.

## Materials and Methods

### Plasmids, antibodies and reagents

The coding sequences for different proteins were PCR amplified from existing plasmids and sub-cloned into respective vectors (Supplementary Table S2). For prokaryotic expression, the coding sequences for Htt-N90_18Q_ and its mutants, and Htt-N171_18Q_ were cloned into a pGEX-4T3 vector. The cDNAs encoding for full-length HSP90 and HSP90(1–696) were cloned into pET-28a, while those for the separate domains of HSP90, including HSP90-N, -M and -C (residues 1–235, 236–548 and 549–696, respectively), HSP90-NM and -MC (residues 1–548 and 236–696), and the mutant (I688A/Y689A/I692A) of HSP90-MC were cloned into pET-22b^+^. The cDNA encoding HSP70 was cloned into pQE-30. For C-terminal GB1-fused Htt-N20 and its mutants (F11A/L14A, A10P, S13P, A10P/S13P), their cDNAs were ligated with that of GB1 and then cloned into pET-22b^38^. For eukaryotic expression, the cDNAs encoding for Htt-N90_100Q_ and its mutants, Htt-N552_18Q_ and Htt-N552_100Q_ were cloned into a FLAG-pcDNA3.1 vector, while those for Htt-N90_18Q_ and its mutants were cloned into pEGFP-N1. The cDNA encoding USP19_b was cloned into an HA-pcDNA3.0 vector^31^. All mutants were generated by site-directed mutagenesis using PCR. All constructs were verified by DNA sequencing. The antibody against FLAG was from Sigma; the anti-HSP90 antibody was from Cell Signaling Technology; the anti-HSP70 antibody was from Proteintech; while the anti-GFP, anti-HA and anti-actin antibodies were from Santa Cruz Biotechnology. All secondary antibodies were purchased from Jackson ImmunoResearch Laboratories. ATP and ADP were purchased from Sigma. The Htt-N20 peptide was obtained from ChinaPeptides Co. by solid-phase synthesis and analyzed by electrospray mass spectrometry.

### Protein expression and purification

Proteins were expressed in *E. coli* BL21 (DE3). The His-tagged proteins were purified by Ni^2+^-NTA column (Roche), and the GST-fused proteins were purified using glutathione Sepharose-4B column (Amersham Biosciences). The ^15^N- and/or ^13^C- labeled Htt-N20-GB1 and its mutants were prepared by using the M9 minimal medium containing ^15^NH_4_Cl and/or ^13^C-glucose as the sole nitrogen and/or carbon resources. All the proteins were further purified by gel filtration chromatography with respective columns (GE Healthcare).

### GST pull-down experiment

The pull-down experiment for GST-Htt-N90_18Q_ or GST-Htt-N171_18Q_ with HSP90 was carried out in a Tris-HCl buffer (50 mM Tris, pH 8.0, 150 mM KCl, 5 mM MgCl_2_, 2 mM DTT, 10% glycerol), while that for GST-Htt-N90_18Q_ or GST-Htt-N90_18Q_M with HSP90 was carried out in another Tris-HCl buffer (50 mM Tris, pH 8.0, 150 mM NaCl, 2 mM DTT, 10% glycerol). The GST or GST-fused proteins were incubated with glutathione Sepharose-4B beads at 4 °C for 1 h, and then HSP90 was incubated with immobilized GST or GST-fused proteins at 4 °C for 2 h. The beads were collected by centrifugation and washed five times with the same buffer, and then eluted by a GSH/Tris-HCl buffer (50 mM Tris, 10 mM GSH, pH 8.0).

### Circular dichroism (CD) spectroscopy

All Far-UV CD spectra were acquired by using a JASCO J-715 spectropolarimeter as described previously^42^. The Htt-N20 peptide was dissolved in a phosphate buffer (100 mM phosphate, 100 mM NaCl, pH7.0) and diluted to the concentration of 0.1, 0.2, 0.5 or 1.0 mg/mL. The cuvette path-length was 1 mm or 0.1 mm when needed. The spectrum of each sample was obtained by averaging three scans on the wavelength from 250 to 190 nm at room temperature. Data were further processed for noise reduction, base-line subtraction and signal averaging when needed, and presented as mean residue molar ellipticity (deg cm^2^/dmol).

### NMR spectroscopy and titration

All NMR data were recorded at 25 °C on a Bruker 600-MHz AVANCE III spectrometer equipped with a TCI CryoProbe proton-optimized triple resonance NMR inverse probe (Bruker). The chemical-shift and NOE assignments for Htt-N20-GB1 were obtained by triple resonance NMR experiments with a ^15^N/^13^C-labeled sample. Data processing and structure calculations were performed following the protocol for NMR structural analysis^43^. ^15^N-labeled proteins (50 μM) dissolved in a phosphate buffer (20 mM phosphate, 50 mM NaCl, pH 6.5) were titrated with HSP90(1–696), its separate domains or HSP70, respectively, and then the [^1^H, ^15^N] HSQC spectra were acquired to monitor the peak intensity changes upon titration. The transferred NOE (TrNOE) experiment was performed as previously described by our laboratory^39^. The Htt-N20 peptide (~1 mM) and HSP90(1–696) (50 μM) were dissolved in a phosphate buffer (20 mM phosphate, 50 mM NaCl, pH 6.5), thus the peptide was 20-fold molar excess to the protein. The 2D ^1^H-^1^H NOESY spectra (mixing time = 200 ms) were recorded to obtain the NOEs of protein-bound and protein-free peptides.

### Size-exclusion chromatography (SEC)

Size exclusion chromatography was performed with Superdex-200 Increase 10/300 GL column on AKTA chromatography system (GE Healthcare). Purified proteins (~100 μM) were loaded on the column in a phosphate buffer (20 mM phosphate, 50 mM NaCl, pH 6.5). A calibration was carried out by using the following standard proteins: Cyt. C (12.4 kDa), Trx (13.9 kDa), OVA (44.3 kDa), GST (52.6 kDa for dimer) and BSA (67.0 kDa). Through plotting of the V_e_/V_o_ (elution volume/void volume) values versus their molecular weights (in logarithm), the apparent molecular weights of the sample proteins were estimated.

### Cell culture, transfection and Western blotting

Human HEK 293 T cells were cultured in DMEM (HyClone) supplemented with 10% fetal bovine serum (Gibco) and penicillin-streptomycin at 37 °C under a humidified atmosphere containing 5% CO_2_. The transient transfection of cells was performed by using PolyJet^TM^ reagent (SignaGen) following the manufacturer’s instructions. Cells were harvested 48 h after transfection and lysed in a RIPA buffer (50 mM Tris, pH 7.5, 150 mM NaCl, 1 mM EDTA, 1% NP-40, and protease-inhibitor mixture (Roche)). The lysates were subjected to SDS-PAGE and transferred onto PVDF membranes (PerkinElmer). The indicated proteins were probed with the respective primary and secondary antibodies and visualized by using an ECL detection kit (Thermo scientific). For quantification and statistical analysis, the band intensity was quantified by using *Scion Image*, and its integral area of gray value was calculated and normalized to that of the control. Data were statistically analyzed with one-way ANOVA.

### Supernatant/pellet fractionation and filter trap experiment

The experimental procedures for supernatant/pellet fractionation and filter trap were following our previous work^31^. Briefly, HEK 293T cells were harvested 72 h after transfection and lysed in 100 μL of a RIPA buffer on ice for 0.5 h, and then centrifuged at 16,200 g for 15 min at 4 °C. The supernatant was added with 100 μL of 2 × loading buffer (4% SDS), while the pellet was sufficiently washed with the RIPA buffer for three times and then added with 40 μL of 4 × loading buffer (8% SDS). The samples were subjected to SDS-PAGE and Western blotting. The filter trap experiment was performed as previously described to detect protein aggregates in cell lysates^65,66^. About 48 h after transfection, the cells were lysed in the RIPA buffer and centrifuged at 12,000 rpm (13,800 × g) for 15 min at 4 °C. The total lysates were added with an equal volume of SDS buffer (4% SDS, 100 mM DTT) and boiled for 5 min at 100 °C. The mixture was centrifuged at 12,000 rpm (13,500 × g) for 5 min and the supernatant was filtered through a cellulose acetate membrane (0.2 μm pore size, Whatman). The membrane was washed twice with 2% SDS and the aggregates retained on the membrane were detected with an anti-FLAG antibody.

## Electronic supplementary material

Supplementary Information
