# Supplementary material for: HSP90 recognizes the N-terminus of huntingtin involved in regulation of huntingtin aggregation by USP19
Source: Sci Rep. 2017 Nov 1;7:14797. doi: 10.1038/s41598-017-13711-7 (PMC5666004; doi:10.1038/s41598-017-13711-7)
Supplement: Supplementary file 1 — Supplementary Information [file 41598_2017_13711_MOESM1_ESM.pdf]

HSP90 recognizes the N-terminus of huntingtin involved in regulation of huntingtin aggregation by USP19

Wen-Tian He[1], Wei Xue[1], Yong-Guang Gao[1], Jun-Ye Hong[1], Hong-Wei Yue[1], Lei-Lei Jiang[1], Hong-Yu Hu[1*]

[1]State Key Laboratory of Molecular Biology, CAS Center for Excellence in Molecular Cell Science, Shanghai Institute of Biochemistry and Cell Biology, Chinese Academy of Sciences; University of Chinese Academy of Sciences. 320 Yueyang Road, Shanghai 200031, P. R. China.

* Correspondence and requests for materials should be addressed to H.-Y H. (email: hyhu@sibcb.ac.cn).

SUBJECT AREAS: MECHANISMS OF DISEASES; PROTEIN AGGREGATION

Running title: Recognition of huntingtin by HSP90

Table S1. Experimental restraints and structural statistics of Htt-N20

Number of experimental restraints	
Total unambiguous distance restraints	150
Intra residual	87
Sequential (| i – j | = 1)	30
Short range (2 ≤ | i – j | ≤ 3)	21
Medium range (4 ≤ | i – j | ≤ 5)	7
Long range (| i – j | > 5)	5
Hydrogen bond restraints	0
Dihedral angle restraints	
φ:	0
ψ:	0
Structure model statistics	
RMSD from experimental restraints	
NOE distances (Å)	0.033 ± 0.006
Dihedral angles (deg.)	0
RMSD from idealized geometry	
Bonds (Å)	0.004 ± 0.000
Angles (deg.)	0.568 ± 0.041
Impropers (deg.)	1.772 ± 0.248
Ramachandran analysis	
Residues in most favored regions (%)	45.3
Residues in additionally allowed regions (%)	45
Residues in generously allowed regions (%)	5.28
Residues in disallowed regions (%)	4.48
Average atomic RMSDs (n=10)	
Backbone atoms (Å)	0.81 ± 0.17
Heavy atoms (Å)	1.92 ± 0.34

The structure was obtained from a sample of the C-terminal GB1-fused peptide (Htt-N20-GB1).

Table S2. List of all the constructs used in this study

Constructs	Vectors	Restriction Enzyme sites	Forward (F) and Reversed (R) Primers
pGEX-4T3-Htt-N90$_{18Q}$	pGEX-4T3	BamHI / XhoI	F: CGCGGATCCATGGCGACCCTGGAAAAG;<br>R: CCGCTCGAGTTATGGTCGGTGCAGCGGCTC
pGEX-4T3-Htt-N90$_{18Q}$M	pGEX-4T3	BamHI / XhoI	F:GCTGATGAAGGCCGCCGAGTCCGCCAAGTCCTTCCAGC;<br>R:GCTGGAAGGACTTGGCGGACTCGGCGGCCTTCATCAGC
pGEX-4T3-Htt-N171$_{18Q}$	pGEX-4T3	BamHI / EcoRI	F: CGCGGATCCATGGCGACCCTGGAAAAG;<br>R: CCGGAATTCTTACTCGAGCTGTAACCTTGG
pET-28a-HSP90	pET-28a	BamHI / XhoI	F: CGCGGATCCATGCCTGAGGAAACCCAG;<br>R: CCGCTCGAGTTAGTCTACTTCTTCCATGCG
pET-28a-HSP90(1-696)	pET-28a	BamHI / XhoI	F: CGCGGATCCATGCCTGAGGAAACCCAG;<br>R: CCGCTCGAGTTACAGACCAAGTTTGATCAT
pET-22b-HSP90-N	pET-22b^{+}	NdeI / XhoI	F: GGGAATTCCATATGCCTGAGGAAACCCAGACC;<br>R: CCGCTCGAGAGCCTCATCATCGCTTAC
pET-22b-HSP90-M	pET-22b^{+}	NdeI / XhoI	F: GGGAATTCCATATGGAAGAAAAGGAAGACAAA;<br>R: CCGCTCGAGGCCTTCTTTGGTGACTGA
pET-22b-HSP90-C	pET-22b^{+}	NdeI / XhoI	F: GGGAATTCCATATGCTGGAACTTCCAGAGGAT;<br>R: CCGCTCGAGCAGACCAAGTTTGATCAT
pET-22b-HSP90-NM	pET-22b^{+}	NdeI / XhoI	F: GGGAATTCCATATGCCTGAGGAAACCCAGACC;<br>R: CCGCTCGAGGCCTTCTTTGGTGACTGA
pET-22b-HSP90-MC	pET-22b^{+}	NdeI / XhoI	F:GGGAATTCCATATGGAAGAAAAGGAAGACAAA;<br>R: CCGCTCGAGCAGACCAAGTTTGATCAT
pET-22b-HSP90-MC^{mut}	pET-22b^{+}	NdeI / XhoI	F:CACATGCTAACAGGGCCGCCAGGATGGCCAAACTTGGTC TCGAG;<br>R:CTCGAGACCAAGTTTGGCCATCCTGGCGGCCCTGTTAGC ATGTG
pQE-30-HSP70	pQE-30	BamHI / HindIII	F: CGCGGATCCATGGCCAAAGCCGCG;<br>R: CCCAAGCTTTTAATCTACCTCCTCAAT
pET-22b-Htt-N20-GB1	pET-22b^{+}	Htt-N20:<br>NdeI / BamHI;<br>GB1:<br>BamHI / XhoI	(Htt-N20) F: GGGAATTCCATATGGCGACCCTGGAAAAG;<br>R: CGCGGATCCCTGCTGCTGGAAGGACTT;<br>(GB1) F: CGCGGATCCCAGTACAAGCTTGCTCTG;<br>R: CCGCTCGAGTTCGGTTACCGTGAAGGT
pET-22b-Htt-N20M-GB1	pET-22b^{+}	Htt-N20M:<br>NdeI / BamHI;<br>GB1:<br>BamHI / XhoI	F: GCTGATGAAGGCCGCCGAGTCCGCCAAGTCCTTCCAGC;<br>R: GCTGGAAGGACTTGGCGGACTCGGCGGCCTTCATCAGC
pET-22b-Htt-N20^{A10P}-GB1	pET-22b^{+}	Htt-N20^{A10P}:<br>NdeI / BamHI;<br>GB1:<br>BamHI / XhoI	F: GAAAAGCTGATGAAGCCATTCGAGTCCCTCAAG;<br>R: CTTGAGGGACTCGAATGGCTTCATCAGCTTTTC
pET-22b-Htt-N20^{S13P}-GB1	pET-22b^{+}	Htt-N20^{S13P}:<br>NdeI / BamHI;	F: GATGAAGGCCTTCGAGCCACTCAAGTCCTTCCAG;

		GB1: BamHI / XhoI	R: CTGGAAGGACTTGAGTGGCTCGAAGGCCTTCATC
pET-22b-Htt-N20$^{A10P/S13P}$-GB1	pET-22b^{+}	Htt-N20$^{A10P/S13P}$: NdeI / BamHI; GB1: BamHI / XhoI	F: GAAAAGCTGATGAAGCCATTCGAGCCACTCAAG; R: CTTGAGTGGCTCGAATGGCTTCATCAGCTTTTC
FLAG-pcDNA3.1-Htt-N90$_{100Q}$	FLAG-pcDNA3.1	BamHI / XbaI	F: CGCGGATCCATGGCGACCCTGGAAAAG; R: GCTCTAGATTATGGTCGGTGCAGCGGCTC
FLAG-pcDNA3.1-Htt-N90$_{100Q}$ M	FLAG-pcDNA3.1	BamHI / XbaI	F: GCTGATGAAGGCCGCCGAGTCCGCCAAGTCCTTCCAGC; R: GCTGGAAGGACTTGGCGGACTCGGCGGCCTTCATCAGC
FLAG-pcDNA3.1-Htt-N90$_{100Q}$ ΔN	FLAG-pcDNA3.1	BamHI / XbaI	F: CGCGGATCCAAGTCCTTCCAGCAGCAG; R: GCTCTAGATTATGGTCGGTGCAGCGGCTC
FLAG-pcDNA3.1-Htt-N552$_{18Q}$	FLAG-pcDNA3.1	BamHI / XbaI	F: CGCGGATCCATGGCGACCCTGGAAAAG; R: GCTCTAGATCAATCATTCAGGTCCATGGC
FLAG-pcDNA3.1-Htt-N552$_{100Q}$	FLAG-pcDNA3.1	BamHI / XbaI	F: CGCGGATCCATGGCGACCCTGGAAAAG; R: GCTCTAGATCAATCATTCAGGTCCATGGC
pEGFP-N1-Htt-N90$_{18Q}$	pEGFP-N1	XhoI / BamHI	F: CCGCTCGAGATGGCGACCCTGGAAAAG; R: CGCGGATCCCGTGGTCGGTGCAGCGGCTC
pEGFP-N1-Htt-N90$_{18Q}$M	pEGFP-N1	XhoI / BamHI	F:GCTGATGAAGGCCGCCGAGTCCGCCAAGTCCTTCCAGC; R:GCTGGAAGGACTTGGCGGACTCGGCGGCCTTCATCAGC
pEGFP-N1-Htt-N90$_{18Q}$ΔN	pEGFP-N1	XhoI / BamHI	F: CCGCTCGAGATGAAGTCCTTCCAGCAGCAG; R: CGCGGATCCCGTGGTCGGTGCAGCGGCTC
HA-pcDNA3.0-USP19_b	HA-pcDNA3.0	EcoRI / XbaI	F: CCGGAATTCACATGTCTGGCGGGGCCAGT; R: GCTCTAGACTAATCCACCTCCTCCATGTT

Figure S1

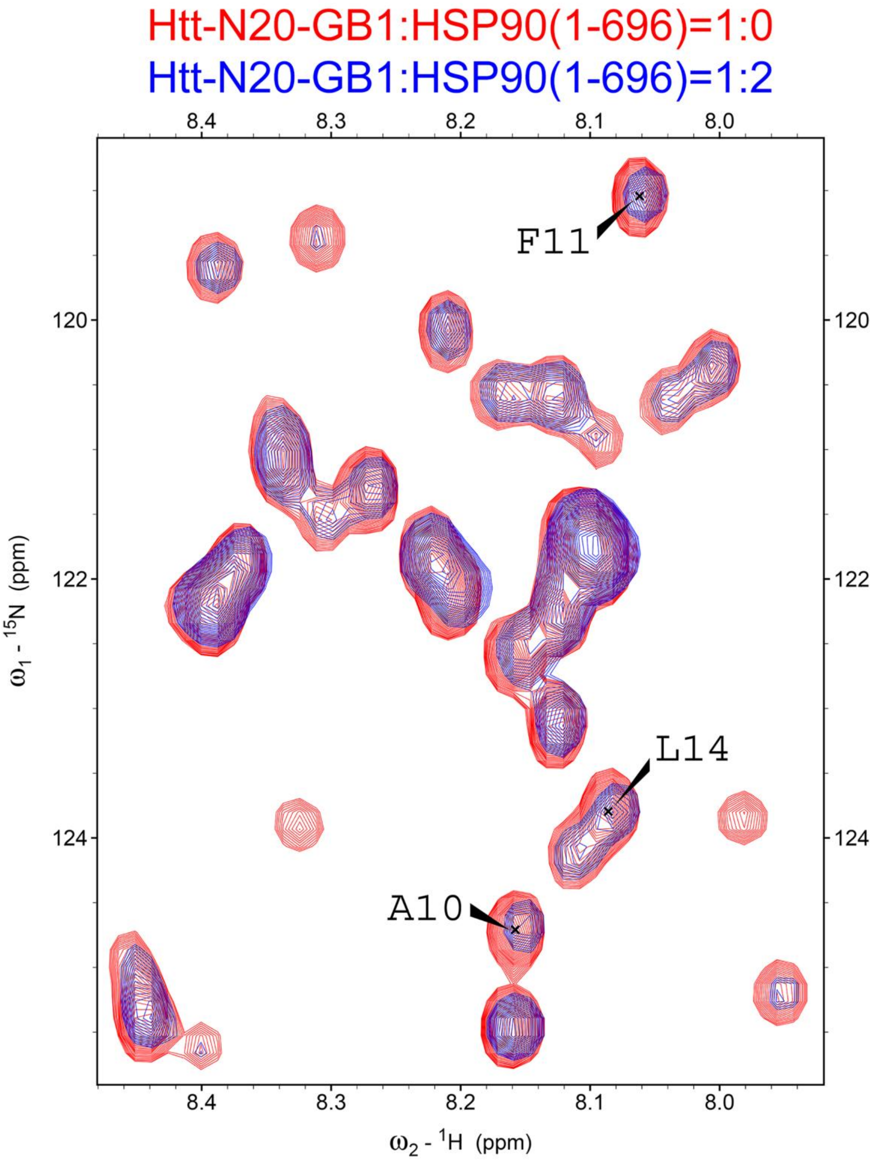

Figure S1. Overlay of the HSQC spectra of Htt-N20-GB1 (red) and titration with HSP90(1-696) (blue). ^{15}N-labeled Htt-N20-GB1 (50 μM) was titrated with HSP90(1-696) at a molar ratio of 1:2. The residues resided in Htt-N20 with an obvious peak intensity change are indicated.

Figure S2

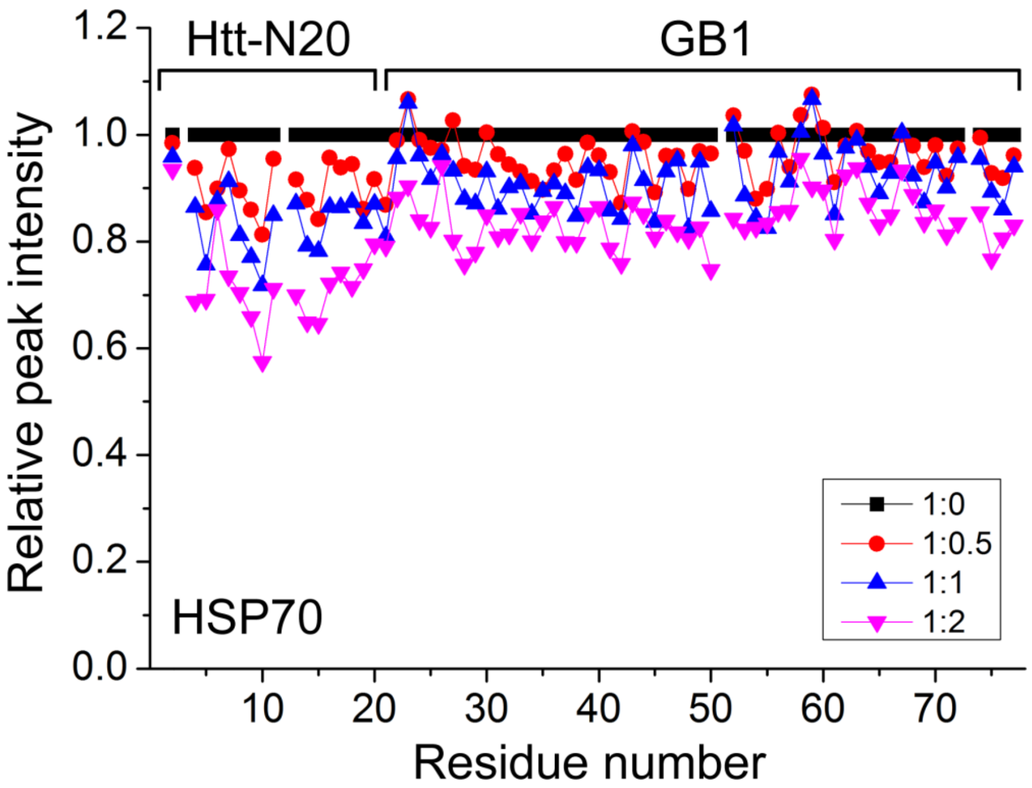

Figure S2. NMR titration showing the interaction between Htt-N20 and HSP70. Plot of the relative peak intensities of amides against the residue number of Htt-N20-GB1 upon titration with HSP70.

Figure S3

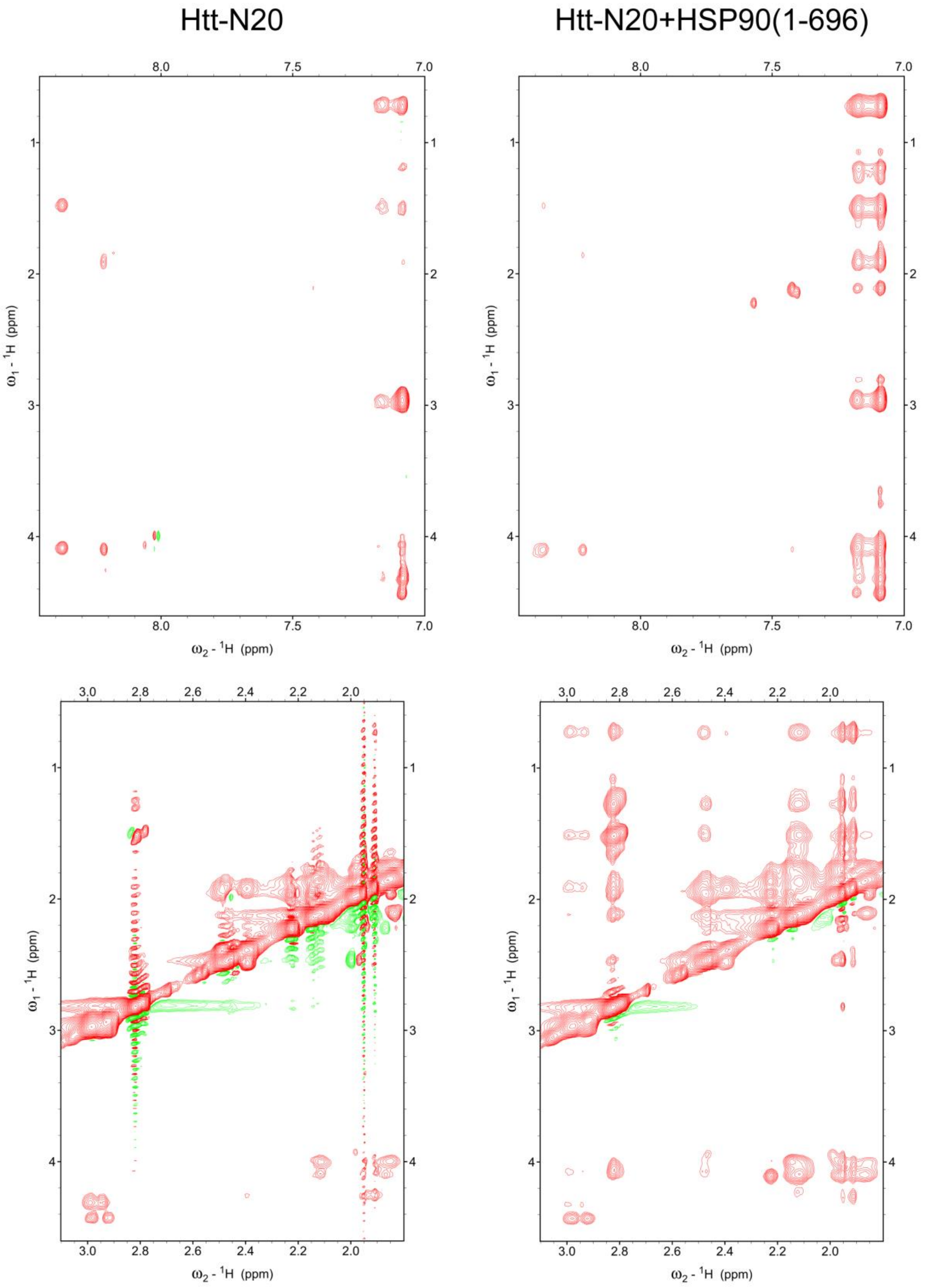

Figure S3. Transferred NOE spectra of the Htt-N20 peptide bound with HSP90(1-696). Representative regions (aliphatic H-NH) of the 2D ^{1}H-^{1}H NOESY spectrum (τ_m = 200 ms) of the Htt-N20 peptide (~1 mM) in the presence of HSP90(1-696) (50 μM) (right panel). The NOESY spectrum (τ_m = 200 ms) of free Htt-N20 showing few NOEs (left panel) was set as a control.

Figure S4

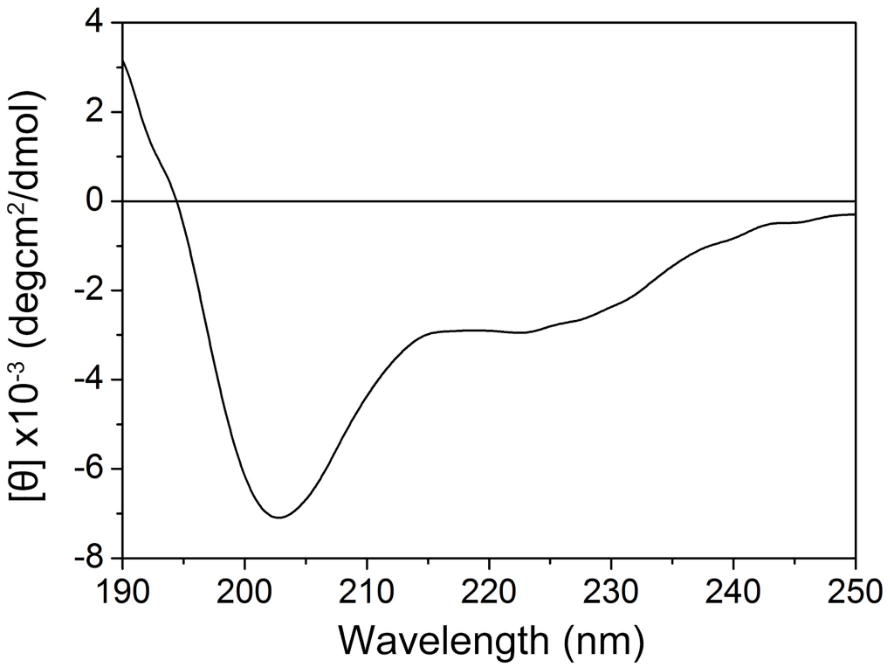

Figure S4. The CD spectrum of Htt-N20 at a concentration of 1.0 mg/mL. To better record the far-UV CD spectrum (250 - 190 nm) of Htt-N20 at high concentration, a cuvette with a path-length of 0.1 mm was used for reducing noise.

Figure S5

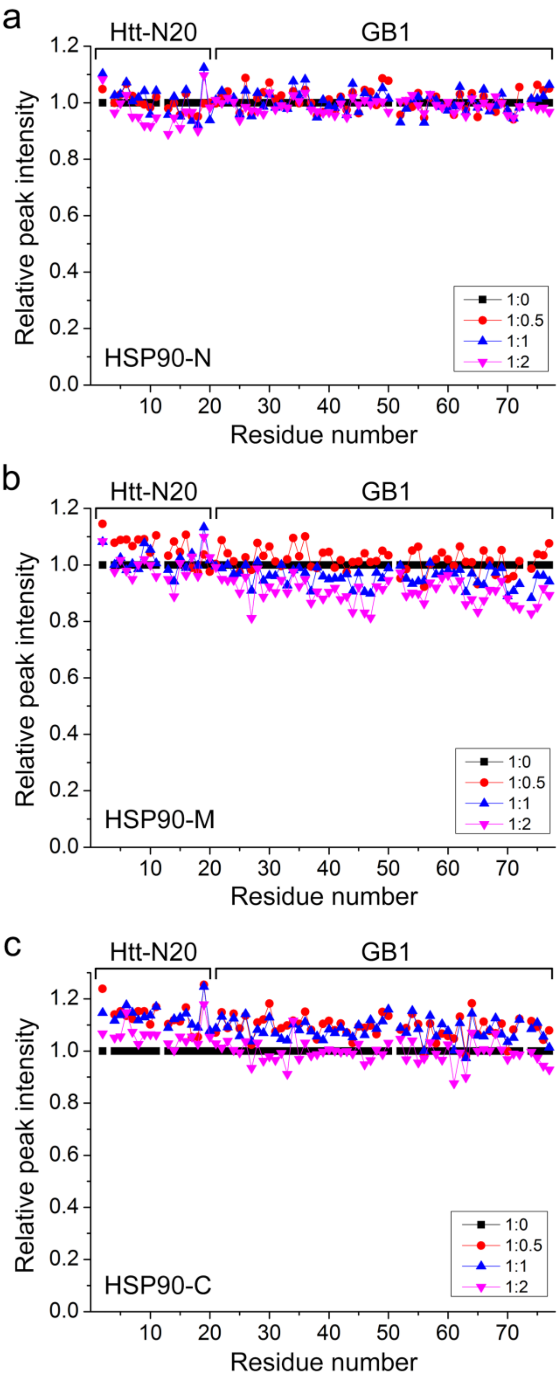

Figure S5. NMR titration of Htt-N20-GB1 with separate domains of HSP90. (**a**) Plot of the relative peak intensities of amides against the residue number of Htt-N20-GB1 upon titration with HSP90-N (residues 1 - 235). (**b**), As in (a), Htt-N20-GB1 with HSP90-M (residues 236 - 548). (**c**), As in (a), Htt-N20-GB1 with HSP90-C (residues 549 - 696). All three separate domains of HSP90 did not bind with Htt-N20.
